# Clinical presentation, diagnosis and management of aerodigestive tract foreign bodies in the adult population: Part 1

**DOI:** 10.4102/sajr.v25i1.2022

**Published:** 2021-03-23

**Authors:** Rishi P. Mathew, Sreekutty Sarasamma, Merin Jose, Ajith Toms, Vinayak Jayaram, Vimal Patel, Gavin Low

**Affiliations:** 1 Department of Radiology, Faculty of Radiology, Rajagiri Hospital, Aluva, India; 2 Department of Radiology & Diagnostic Imaging, Faculty of Medicine and Dentistry, University of Alberta Hospital, Edmonton, Canada

**Keywords:** foreign body, fish bone, dentures, food bolus, bezoar, body packing

## Abstract

In the adult population, foreign bodies may be accidentally or intentionally ingested or even inserted into a body cavity. The majority of accidentally ingested foreign bodies pass through the alimentary tract without any complications and rarely require intervention. Accidentally ingested foreign bodies are usually fish bones, bones of other animals, and dentures. Oesophageal food impaction is the commonest cause of oesophageal foreign bodies in the Western hemisphere. Intentionally ingested foreign bodies may be organic or inorganic, and often require intervention; these patients have either underlying psychological or mental disease or are involved in illegal activities such as body packing, which involves trafficking narcotics. Imaging plays a crucial role in not only identifying the type, number and location of the foreign body but also in excluding any complications. In this comprehensive pictorial review, we provide an overview of the spectrum of foreign bodies ingested in adults, emphasising the role of various imaging modalities, their limitations and common foreign body mimickers on imaging.

## Introduction

Foreign body ingestion is a common clinical presentation with approximately 80% – 90% of all ingested foreign bodies passing through the digestive tract without need for intervention. Only about 10% – 20% require endoscopic removal and hardly 1% need surgery. However, the statistics change when it comes to intentionally ingested foreign bodies, with nearly 76% of the cases requiring non-surgical intervention and 28% needing surgery.^1^

Clinical symptoms may be acute, including epigastric pain, vomiting, dysphagia, pharyngeal discomfort and chest pain. Interestingly, 30% of the patients may be asymptomatic, even for years.^1^

The intention of this article is to inform readers of the risk factors, the common locations where foreign bodies become lodged in the aerodigestive tract, useful imaging modalities and how to protocol them to aid in the diagnosis, how to identify the complications when present and the common foreign body mimickers on imaging.

## Discussion

Foreign bodies may be ingested, inserted intentionally into a body cavity or accidentally deposited by trauma or iatrogenic injury. Most ingested foreign bodies pass naturally, with nearly 80% having a benign course. Approximately 1500 people die annually in the United States from foreign body ingestion.^1,2^ Four broad categories of patients can present with foreign body ingestion or insertion, namely: (1) children, (2) mentally challenged persons, (3) adults with unusual sexual behaviours and (4) adults or children with pre-existing factors or injurious situational conditions (e.g. drug and/or alcohol abuse, extreme sports, criminal offenders, and those prone to child or spousal abuse). Mentally challenged individuals are repeat offenders, presenting multiple times with foreign body insertion or ingestion. Foreign body impaction in adults generally results from predisposing conditions such as strictures (37%), malignancy (10%), oesophageal rings (6%) and achalasia (2%).^3,4^

### Accidentally ingested foreign bodies

As per published data, the foreign bodies most commonly swallowed by adults are fish bones (9% – 45%), bones of other animals (8% – 40%) and dentures (4% – 18%).^4,5^

#### Fish bone ingestion

Accidental fish bone ingestion is a commonly encountered problem in the emergency department, especially across Asia and the Mediterranean where ingestion of unfilleted fish occurs regularly.^6,7,8^ Because of the higher consumption of fish in Asian countries, fish bones can account for as high as 60% of all accidentally ingested foreign bodies in that part of the world.^9^

The most common predisposing or risk factor for accidental fish bone ingestion is the use of dentures. Dentures impair the natural feedback of the palatal sensory nerves that are required to identify sharp and hard textured contents in a food bolus. Other less common factors are rapid eating, talking whilst eating, alcoholism and mental retardation.^10^

**FIGURE 1:**
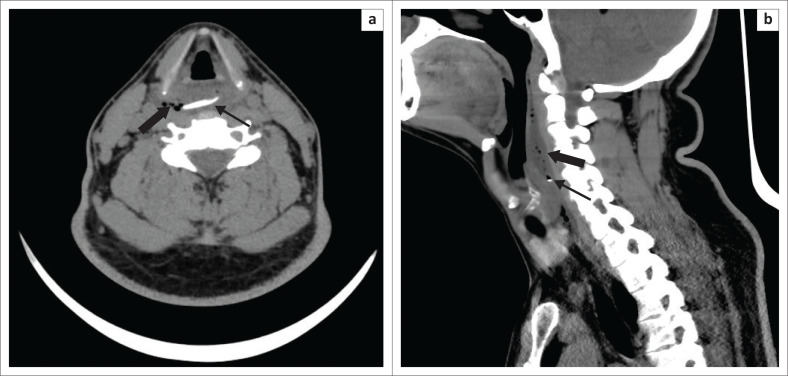
Impacted fish bone in a 45-year-old male. Frontal and lateral radiographs of the neck (a and b) show no radio-opaque foreign body. Sagittal (c) and coronal (d) reformatted computed tomography images demonstrate the fish bone (arrows) impacted in the left lateral pharyngeal wall.

**FIGURE 2:**
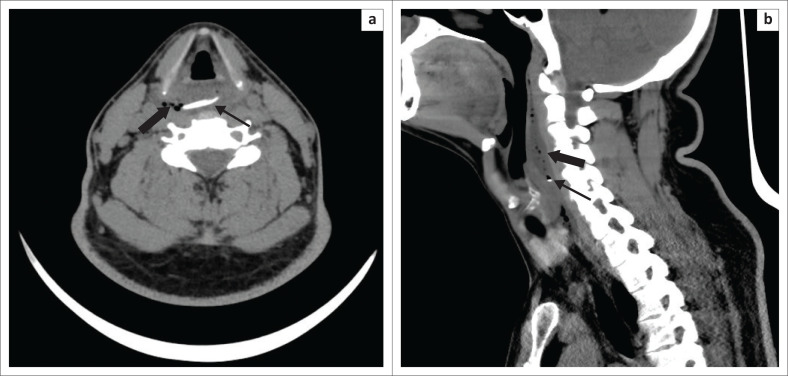
Axial (a) and sagittal reformatted (b) computed tomography images showing a 4 cm × 1 cm fish bone (thin arrows) impacted in the posterior cricopharyngeal wall with perforation resulting in a retropharyngeal abscess (thick arrow).

Once swallowed, fish bones usually become lodged in the oral cavity or pharynx (Figure 1a–d), especially in the tonsils, at the tongue base, vallecula or pyriform fossa, with the other less common sites of impaction being the oesophagus, stomach, small bowel and colon.^9,10,11^ Uncomplicated cases of impaction in the oropharynx can be easily visualised by an Ear Nose Throat (ENT) surgeon and removed using a scope.^11^ Within the oesophagus, the most common site of fish bone impaction is in the cervical portion, in the cricopharyngeus muscle at the C5–C6 level (Figure 2a, b), followed by the thoracic portion at the level of the aortic arch; uncommon sites are the left subclavian artery origin or at the origin of an aberrant right retroesophageal artery.^10,11^

Foreign body aspiration into the tracheo-bronchial tree is rare in adults, and when present the fish bone may be seen in the right bronchial tree because of the anatomical, near parallel orientation of the right main bronchus with the trachea. These patients may be asymptomatic or can present with a cough.^11^

The value of symptoms (e.g. foreign body sensation, dysphagia, odynophagia, vomiting, blood-stained saliva, retrosternal pain, etc.) in locating the fish bone site remains controversial. Foreign body sensation is the only symptom found to correlate with the site of lodgement, with a high success of retrieval in the proximal oesophagus (59%) in comparison to the lower oesophagus (11%). Laterality of symptoms may also indicate an ipsilateral location of the foreign body, as reported by Klein et al.^12^

Conventional neck radiography (lateral view), being inexpensive and widely available, is often the first imaging modality used for evaluating patients who have accidentally ingested a fish bone. However, its diagnostic utility in identifying fish bones remains questionable and controversial with a reported false negative rate of 47%^12^ and a very low sensitivity of 25.3%.^12,13^ Factors that affect the detection of foreign bodies on the lateral view neck radiograph are location of impaction, orientation and the density of the foreign body. A fish bone may be impacted anywhere in the aerodigestive tract, and the surrounding normal soft tissue density may hinder its detection, especially at the level of the cricopharyngeus muscle. In terms of orientation, a foreign body oriented orthogonal to the radiograph is much easier to identify than one that is oriented horizontally. The optical density of the bones of various fish species differ, making it difficult to identify at radiography.^13^ In addition, normal anatomical structures can often mimic an ingested foreign body on the lateral neck radiograph and these include partial ossification of the superior cornu of the thyroid cartilage, arytenoid cartilage, posterior lamina of the cricoid cartilage, the stylohyoid and thyrohyoid ligaments, the styloid processes and vascular calcifications. Knowledge of the clinical history, typical anatomical landmarks on the neck radiograph and the lack of ancillary findings such as prevertebral soft tissue swelling or cervical emphysema can help differentiate these common mimickers from an impacted foreign body.^13^

**FIGURE 3:**
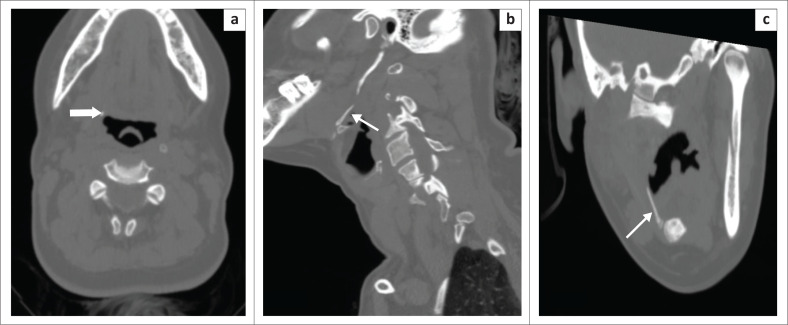
(a–c) Computed tomography images of a 65-year-old woman with a foreign body sensation in the right neck following a meal: (a) Axial image shows suspicious hyperdense material near the base of the tongue (arrow) on the right side, mimicking a foreign body. However, (b) sagittal reformatted and (c) 3D multiplanar reformation (MPR) images confirmed it to be the greater cornu or horn of the hyoid bone.

Multidetector computed tomography (MDCT) is highly sensitive and specific for identifying foreign bodies, with the overall reported sensitivity and specificity being in the range of 90% – 100% and 93.7% – 100%, respectively.^13^ Potential pitfalls on MDCT include tonsilloliths (that appear as tiny, rounded structures with well-defined margins, unlike foreign bodies that are usually linear or irregular in shape), the hyoid bone (Figure 3a, b), cricoid calcifications, swallowing motion artefacts (that appear streaky in density) and artefacts from radiodense materials, for example, barium or silver nitrate, or even faecal material in the bowel^11,13^ and slice thickness.^14^ A major limitation for the detection of foreign bodies on computed tomography (CT) is the lack of observer awareness.^14^ The use of contrast (oral or intravenous [i.v.]) can hamper the identification of fish bones on MDCT. Oral contrast can conceal fish bones in the intestinal lumen, whilst extraluminal fish bones can mimic blood vessels on i.v. contrast studies.^14^ If there is a strong clinical suspicion for accidental fish bone ingestion and if the initial study is an i.v. contrast examination that was negative, then the study needs to be repeated without contrast. Factors that can improve detection on MDCT include the use of thinner reconstructions (3 mm/1.5 mm) and using multiplanar reformatted images for evaluation.^10,14^

**FIGURE 4:**
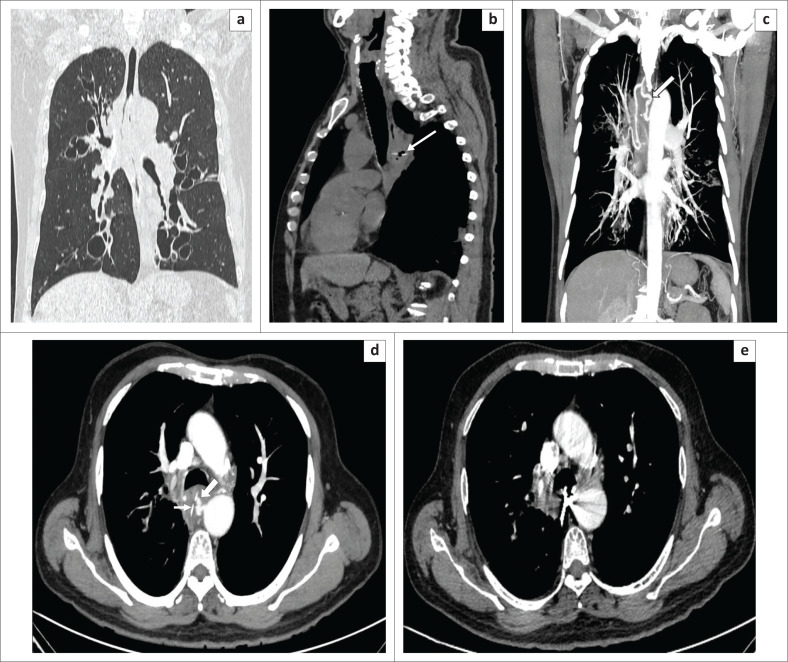
Computed tomography (CT) images of a 61-year-old man with a pseudoaneurysm of a hypertrophied bronchial artery following impaction of a retro-oesophageal fish bone (FB). Reformatted coronal CT images indicate the (a) underlying bronchiectasis (b) impacted retroesophageal FB and collection (thin arrow). (c) Post contrast 3D-multiplanar reformation (MPR) coronal image shows the hypertrophied bronchial artery (thick arrow). (d) Post contrast 3D CT axial image shows the impacted FB (thin arrow) causing a pseudoaneurysm of the hypertrophied bronchial artery (thick arrow). (e) Post embolisation of the bronchial artery pseudoaneurysm; the FB was removed.

Complications associated with accidentally ingested fish bones are uncommon and when present are usually laceration and penetration or perforation of the pharyngoesophageal wall.^6,15^ Pharyngeal fish bone impaction can cause infection of the deep neck space, neck abscess and retropharyngeal haematoma or abscess formation. Migrated soft tissue fish bones can cause a retropharyngeal abscess, oesophageal dissection, penetration of the facial artery or even the parotid duct. In severe cases it can even damage the cardiovascular system, causing complications such as pericarditis, cardiac tamponade, infectious endocarditis, systemic air embolism, pseudoaneurysm (Figure 4a, e) or aorto-oesophageal fistula.^12,16^ Perforations below the level of cricopharyngeus are uncommon,^10,17^ and when present the common sites of perforation are usually the lesser curvature of the stomach^18^ and the less mobile portions or regions with acute angulations in the bowel, such as the ileum and the rectosigmoid junction.^11,19^ Pneumoperitoneum is rare with fish bone impaction, as bowel perforation occurs by slow erosion through the wall, which is spontaneously sealed by fibrin and omentum.^11,19^

#### Chicken bone and other bone fragments

**FIGURE 5:**
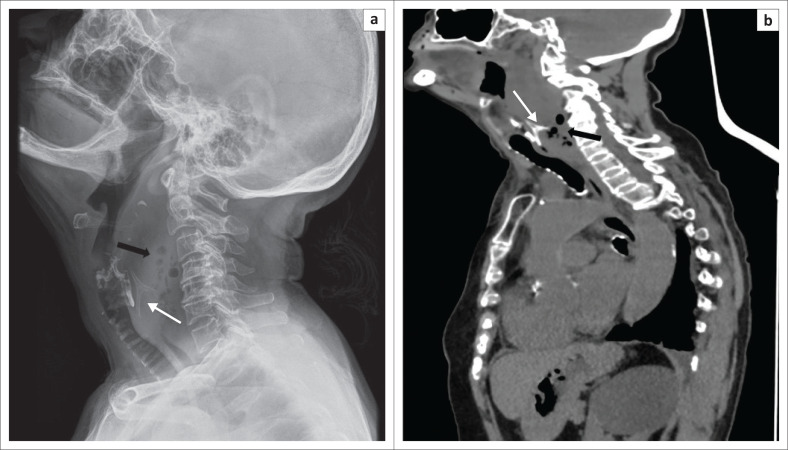
(a) Lateral radiograph of the neck shows a ‘Y’ shaped chicken bone (thin arrow) impacted in the posterior wall of the cricopharynx, along with prevertebral soft tissue thickening demonstrating multiple air locules (thick arrow). (b) Computed tomography sagittal reformatted image confirmed the chicken bone (thin arrow) location and the retropharyngeal abscess.

When compared with fish, the bones of other animals (e.g. chicken, beef, etc.) tend to be more radio dense. A lateral neck radiograph can often localise an impacted chicken bone in the neck (Figure 5a). The ability of a radiograph to satisfactorily demonstrate an ingested chicken bone depends on the radio-opacity of the bone and image quality of the radiograph. However, a CT will be necessary to assess for complications such as perforation and abscess formation (Figure 5b).^20^

#### Dentures

Dentures are medical prosthetic devices used for improving aesthetics, mastication, articulation and self-esteem. Ingestion of dentures is rare amongst the young and healthy population and is more common amongst the elderly, in alcohol or drug abusers and patients with psychoneurologic deficit. An additional risk factor is the dislodgment or loosening of removable or fixed dentures.

The most common site for denture impaction is in the oesophagus (70%). Small bowel impaction is rare, and when present, is usually in the terminal ileum. Complications associated with denture impaction include perforation (most common), necrosis, penetration to adjacent organs, haemorrhage and bowel obstruction.^21^

**FIGURE 6:**
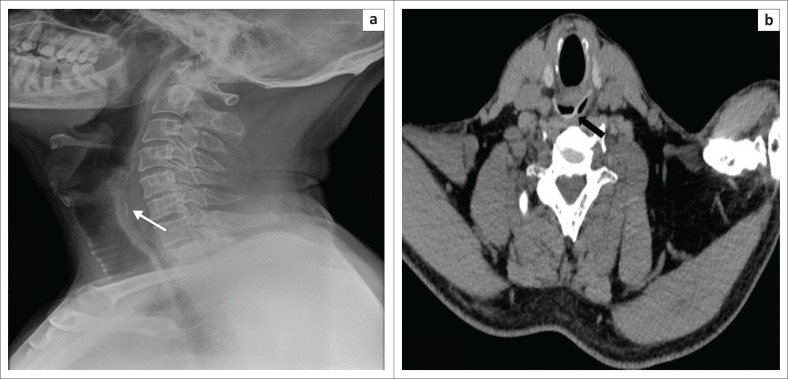
(a) Lateral radiograph of the neck revealing a swallowed denture (thin arrow) at the level of the cricopharynx. (b) Axial computed tomography image confirms the location of the denture (thick arrow), which appears as a curvilinear hyperdense structure.

Soft tissue lateral radiographs of the neck are routinely performed in patients who have ingested dentures. However, its clinical value in these cases is questionable as the dental plates are radiolucent (unlike natural teeth, which can be visualised on plain radiographs).^21,22^ This has been the case since the 1940s when radio-opaque vulcanite was replaced by radiolucent acrylic materials in dentures. A normal lateral radiograph of the neck, therefore, does not exclude pathology, and an urgent ENT consultation is required in the presence of dysphagia, pain or discomfort in the throat and pooling or excessive production of saliva. Metal components in the dentures such as connectors, clasps, wire retainers or a metal core may allow the denture to be localised on a radiograph.^21,22^ Acrylic dentures are visualised on CT (Figure 6a, b) and on magnetic resonance imaging (MRI); however, limitations to MRI access in an Emergency Room (ER) setting poses a challenge.^22^ A barium contrast study is not recommended and is rarely helpful, as it will coat all sides of a radiolucent object in addition to hampering endoluminal visualisation on subsequent endoscopy.^23^

#### Oesophageal food bolus impaction

Unlike in Asia, oesophageal food impaction is the commonest cause of oesophageal foreign bodies in the Western hemisphere,^6^ with an estimated annual incidence rate of 13 per 100 000.^6,24^ Steakhouse syndrome is the clinical condition resulting from oesophageal food impaction after eating a piece of food (usually a meat bolus) because of inadequate chewing.^25^

Previous studies have shown that 88% – 97% of oesophageal food bolus impaction cases have underlying oesophageal pathologies. Common causes include oesophageal stenosis due to Schatzki rings or peptic strictures, oesophageal webs, extrinsic compression, surgical anastomosis, oesophagitis and achalasia. Eosinophilic oesophagitis is recognised as an emerging cause of food bolus impaction in younger patients.

If the food gets impacted at the upper oesophageal sphincter, it can be readily localised by the patient; however, symptoms occur when the food becomes lodged in the distal oesophagus. Symptoms include neck/chest pain or pressure, dysphagia, odynophagia, a sense of choking, retching and vomiting. Patients with high grade food bolus impaction can present with hypersalivation and inability to swallow any liquids, including their own saliva. Respiratory symptoms develop when there is aspiration of saliva or food from compression of the trachea or complete airway occlusion. On presentation at the ER, clinical examination should assess the patient’s stability and assess for any complications. Initial examination should assess ventilation, airway compromise and the risk for aspiration. Signs of oesophageal perforation are fever, tachycardia, subcutaneous crepitus and neck or chest swelling. Oesophageal perforation requires urgent surgical intervention without any delay.^26^

**FIGURE 7:**
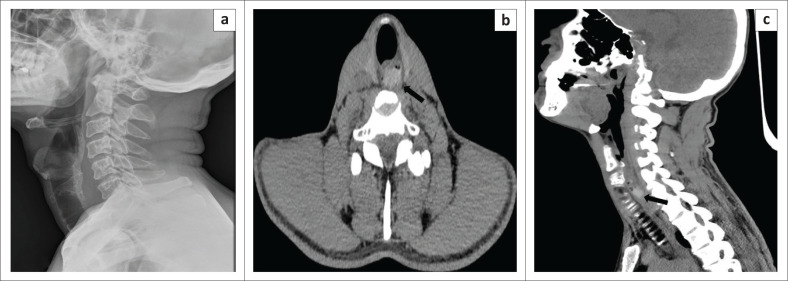
Food bolus (meat) impaction in a 70-year-old male. (a) Lateral radiograph of the neck was unremarkable. Computed tomography (b) axial and (c) sagittal reformatted images shows the impacted bolus of food (black arrow) in the hypopharynx at the C6–C7 level.

Radiographic evaluation can be useful to identify any complications such as pneumomediastinum. Food boluses are radiolucent (Figure 7a–c) and are, therefore, not seen on conventional radiographs. Due to the risk of aspiration, an oral contrast examination should not be performed. Most cases of oesophageal food impaction are treated by flexible endoscopy, which should not be delayed by more than 24 h after presentation because of the risk of complications.^26^

### Intentionally ingested foreign bodies

**FIGURE 8:**
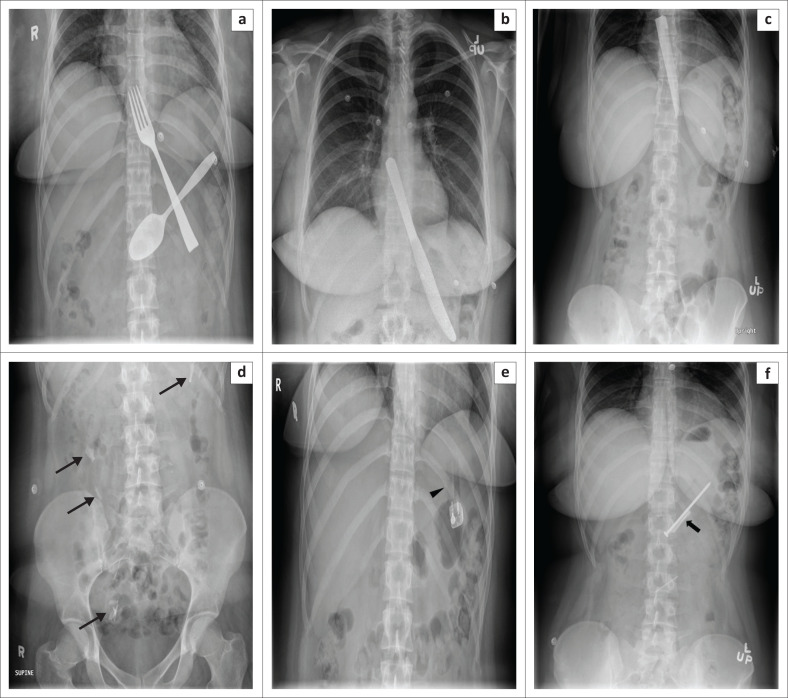
Serial chest and abdominal radiographs of a 25-year-old young woman taken over a period of one year with a history of physical and sexual abuse. The images demonstrate various intentionally ingested foreign bodies that included (a) a fork and spoon, (b) a table knife, and (c) a serrated knife, as well as various intentionally ingested foreign bodies that included (d) multiple glass pieces (thin arrows), (e) a cigarette lighter (arrowhead), and (f) a nail and a razor blade (thick arrow).

Patients who intentionally ingest foreign bodies can be challenging to treat, as the history provided is often confusing or incomplete, making it difficult for the endoscopist to plan the procedure and the type of anaesthesia to be administered. Self-injurious behaviour is common amongst patients with post-traumatic stress disorder (PTSD), psychotic and personality disorders. Often these patients have an underlying history of childhood deprivation, physical and/or sexual abuse (Figure 8a–f).

The pattern of self-injury in these patients is usually non-suicidal and tends to be parasuicidal in nature. It is believed that the self-injurious harm is a form of expression of rage or punishment towards oneself and/or towards their caregivers, or a way to persuade others to provide attention or care. It is believed that these patients feel a sense of empowerment by being able to exasperate or infuriate and challenge the treating physician or surgeon and hence feel motivated to indulge in further ingestions.^27^ Intentional ingestion of foreign bodies can also be seen in Munchausen syndrome or in prison inmates as an act of malingering.^28^

In a study conducted by Huang et al.^29^ involving 305 cases of intentionally ingested foreign bodies, it was found that the average time period from ingestion to presentation was more than 48 h. The most common items ingested were pens, batteries, knives, razor blades, pencils, toothbrushes, spoons, coins and metallic objects, and the overall success rate of endoscopic removal was 90%.

#### Bezoar

**FIGURE 9:**
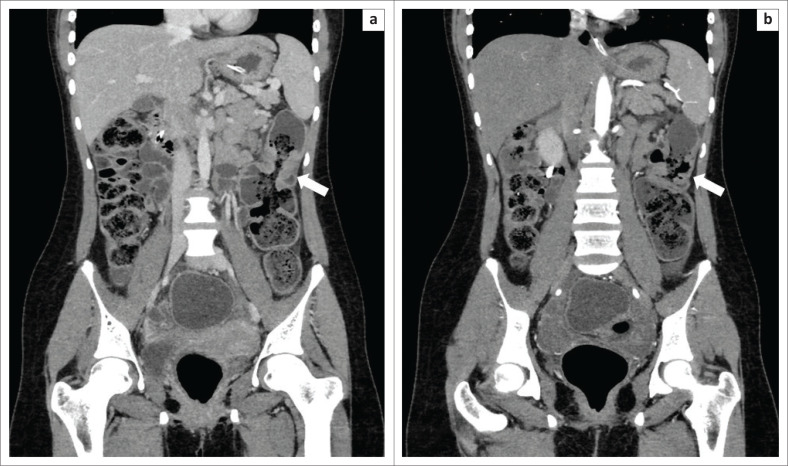
(a and b) Coronal reformatted computed tomography images of a 19-year-old female with a history of repeatedly chewing her ‘dupatta’ (a shawl-like scarf traditionally worn by women of the Indian subcontinent) showing a short segment of bowel wall thickening in the proximal descending colon (arrows). The ‘faecal like’ material within the lumen of the affected descending colon was identified at laparoscopy as textile bezoar.

The term ‘bezoar’ is derived from the Arabic word ‘bedzehr’ or the Persian word ‘padzhar’, meaning ‘protection against a poison’, as in the past bezoars from animal guts were used as antidotes to poisons and still form part of traditional Chinese medicines.^30^ Bezoar refers to a conglomerate mass composed of foreign bodies which, undigested by gastric acid, accumulates within the alimentary canal, most commonly in the stomach (Figure 9a, b). The majority of gastric bezoars result as a complication of gastric surgery. However, gastric bezoars can also occur in the normal stomach from ingestion of various objects that do not easily pass through the gastric pylorus such as hair, prune, plastic, paper, cotton, et cetera.

Phytobezoars and trichobezoars are the commonest forms of bezoars,^31,32^ with the former accounting for nearly 40% of all cases.^32^ It is made up of poorly digested fruits (e.g. oranges, persimmons, etc.) and/or vegetable fibres. A trichobezoar forms the second most common group and is composed of hair, either from the patient, other humans or animals. It is an occupational hazard amongst brush makers, blanket weavers and wool workers. It is most commonly seen in young women and usually located in the stomach; it may rarely migrate into the small bowel.^31,32,33^

**FIGURE 10:**
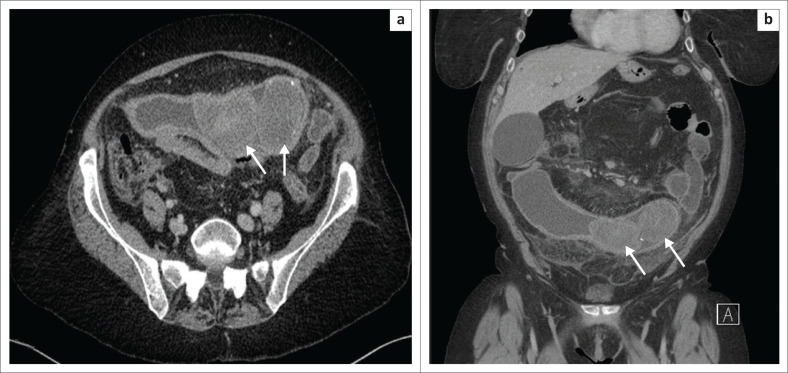
(a) Axial and (b) coronal reformatted computed tomography images of a 64-year-old woman with a history of Crohn’s disease. Computed tomography showed two intraluminal masses (arrows) with peripheral calcifications located within a dilated loop of distal jejunum. There was associated upstream small bowel obstruction. The patient underwent small bowel resection with primary anastomosis, and pathological evaluation of the excised specimen confirmed the intraluminal masses as bezoars.

The condition is most commonly associated with psychiatric conditions such as trichotillomania (an urge to pull one’s own hair) and trichophagia (an urge to eat one’s own hair).^34^ Trichotillomania may also be seen in some neurodevelopmental disorders such as Lesch–Nyhan syndrome, which can be associated with other body-focused repetitive behaviours such as skin picking, scratching, nail, hand or finger biting, head banging, self-hitting, et cetera.^35^ A Rapunzel syndrome is a rare form of gastric trichobezoar with a long tail extending beyond the stomach along the duodenum into the small intestine.^30,32,34,36^ Small bowel obstruction is the most common clinical complication of a bezoar (Figure 10a, b); however, it is responsible for only 0.4% – 4% of all intestinal obstructions.

Until recently, bezoars were rarely diagnosed prior to surgery, as the patient’s symptoms and radiographic findings appeared similar to small bowel obstruction attributable to other causes. However, recent studies have shown that ultrasound or CT can help diagnose a bezoar prior to surgery. Conventional radiographs can help identify complications such as small bowel obstruction. A bezoar is rarely detected by abdominal radiography and can be mistaken for an abscess or the presence of faeces in the colon. Ultrasound may have limitations in those cases of small bowel obstruction where the bezoar is far from the abdominal surface, cases with multiple bezoars, and in patients with gastric bezoars. Gastric bezoars need to be differentiated from ingested food; however, on ultrasound a gastric bezoar causes diffuse posterior acoustic shadowing with an echogenic band that helps to differentiate it from the ‘dirty’ shadowing caused by ingested gas and food within the stomach.^37^ Sonographic features of a bezoar include the presence of an intraluminal bowel mass with an echogenic arch-like surface and profuse acoustic shadowing. The presence of twinkling artefacts within the arch-like surface on colour Doppler may also help to increase the diagnostic confidence. This artefact is because of a narrow band of intrinsic noise within the Doppler circuitry of the ultrasound machine, called phase jitter. Major differentials for a small bowel bezoar on ultrasound are gall stone ileus, small bowel tumours with calcifications and faecal material.^37,38^ It is not common for faecal material to present on ultrasound with a rough surface demonstrating twinkling artefacts and posterior shadowing. However, unlike bezoars, faecal material has a relatively soft consistency and is mobile with dynamic ultrasound imaging.^39^

At CT imaging, a bezoar appears as a well-defined oval intraluminal mass containing air bubbles within the interstices, along with dilated proximal small bowel loops and normal or collapsed distal bowel. An inconsistency between CT and the surgical location of a bezoar is usually because of migration of the bezoar during the interval between the imaging study and surgery. Computed tomography may help to differentiate a small gastric bezoar from food contents, as the former is often round or oval in shape, tends to float on the water–air surface surrounded by gastric contents and is of lower density than food particles; large bezoars tend to occupy all of the lumen, demonstrating diffuse air bubbles throughout the mass. Gastric bezoars may be missed on standard abdominal soft tissue window settings (level 40, width 350 Hounsfield Units [HU]), and the sensitivity may be increased by reducing the window level to approximately -100 HU.^37,39^ On a CT study, small bowel faeces can mimic a bezoar. The former tends to be located proximal to the site of obstruction, unlike bezoars which are located at the site of obstruction. Also, small bowel faeces tend to be more tubular in shape, whilst the latter is often round or oval. One study pointed out that the length of the faeces-like material in the dilated small bowel proximal to the transition zone is a key imaging feature to differentiate small bowel faeces from a bezoar.^40^

Treatment strategies for gastric phytobezoars include chemical dissolution by Coca-Cola^®^, endoscopic removal, laparotomy and laparoscopic surgery. However, phytobezoars caused by persimmon are resistant to chemical dissolution because of their hard consistency and are usually removed by endoscopy or surgery. Intestinal bezoars are commonly removed by surgery as these patients generally present with intestinal obstruction and ileus. Trichobezoars, because of their high intrinsic density, tend to be resistant to enzymatic degradation, pharmacotherapy and endoscopic fragmentation, often requiring laparotomy or laparoscopic surgery.^41^

#### Body packing

Body packing was first reported in the literature in 1973 by Canadian doctors, Deitel and Syed, who presented a case of ileus secondary to ingestion of a condom filled with Hashish.^42^ The term ‘body packing’ refers to ingesting a large amount of narcotic material (usually cocaine, heroin or cannabis products) wrapped in a number of packages so that it can be concealed in the alimentary tract and transported to the target destination (usually across international borders) without being caught by security officials. The individuals involved in this act are called body packers; other names include drug mules, swallowers, intestinal carriers or couriers. The term ‘body stuffer’ or ‘mini packer’ refers to an individual who swallows small amounts of loosely wrapped illicit drugs, plastic pouches or small pellets on an unexpected encounter with law enforcement officials for fear of being arrested. The term ‘body pusher’ refers to individuals who insert narcotic packages into their rectum or vagina.^43^ As a result of the limitation of this article, we will not be discussing the latter group. The packing materials used for these illicit drugs may be handmade or manufactured. Commonly used synthetic materials include condoms, plastic wraps or bags, latex glove fingers, balloons, aluminium foil, cellophane and glassine. These packages are also known as bolitas. In the majority of the cases, the narcotics are usually solid drugs; however, recently a novel method of smuggling has emerged where the body packers ingest packages stuffed with liquid cocaine.^43,44^

**FIGURE 11:**
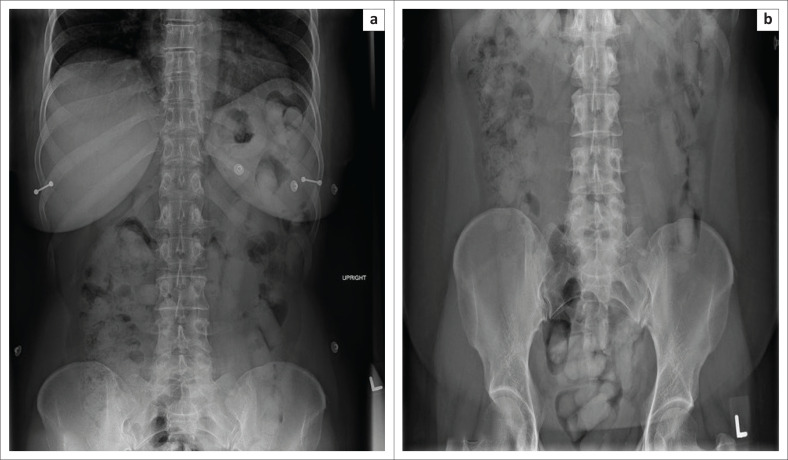
(a and b) Abdominal radiographs of packages containing narcotic materials in a 45-year-old woman.

The various imaging modalities available for identifying body packing are plain abdominal radiography, ultrasound, CT and MRI. Of these, the first two are the most commonly used screening tools, with plain abdominal radiography being the most widely used test because of its lower cost and ease of availability. The reported sensitivity for plain abdominal radiography in body packing is between 40% and 90%.^43,44^ The presence of one or more well-defined opacities in the stomach, small or large intestine, that are not suggestive of gastrointestinal contents, should raise suspicion for body packing. Other ancillary imaging findings or signs that have been described are: (1) the ‘double condom sign’ – the presence of radiolucent air crescent trapped between multiple layers of packing surrounding each packet; (2) the ‘tic-tac’ or ‘bag of eggs’ *sign* – refers to the presence of several homogeneous radiodense oval or round shaped structures with sharp margins and clear air-substance interface (Figure 11a); (3) the ‘parallelism sign’ – firm narcotic packs aligning parallel to each other in the intestinal lumen; (4) the ‘rosette sign’ – refers to air trapped in the knot of the drug packet; (5) the ‘halo sign’ – a complete rim of blurred lucency surrounding the drug packet^43,44^; (6) the ‘black crescent sign’ – a crescent of air around the drug packet; and (7) the ‘lucent triangle sign’ – representing air in the interface between drug packets or with the bowel wall. The ‘double condom’ and ‘halo’ signs that have traditionally been used for detecting packets containing solid drugs, are also effective in detecting liquid cocaine. In addition, packets with liquid narcotics tend to be irregular, with ill-defined borders and show variable density unlike solid drugs that are often opaque to faeces (Figure 11b).^44^ Common mimickers for drug packets on conventional radiography include hardened faeces (faecaloma or scybala), intestinal air, calcifications and other foreign materials.^43,44,45^

On ultrasound, packages containing solid drugs appear as multiple, ovoid, immobile, smooth contoured echogenic masses within the bowel lumen, demonstrating strong posterior acoustic shadowing. Moreover, reverberation artefacts may be seen behind the packets secondary to trapped air in the sheaths. However, ultrasound has limitations in differentiating packets containing liquid narcotics from intestinal gas and faeces.^45^

**FIGURE 12:**
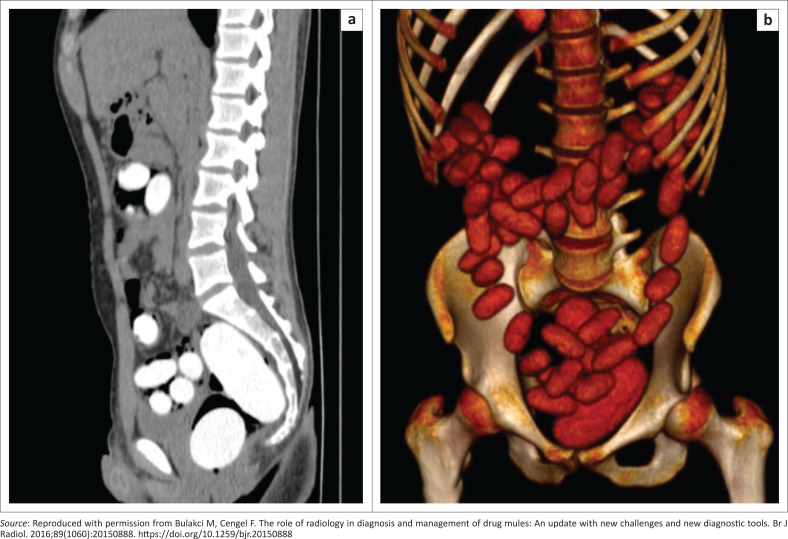
A 25-year-old male body packer and body pusher. (a) Sagittal computed tomography imaging of the intestinal loop lumens without contrast material revealed multiple hyperdense drug-filled capsules along with two bigger sized packages in the lumen of the rectum. (b) Three-dimensional volume-rendering technique images distinguish the capsules more easily. A total amount of 1093 g of 60 cocaine capsules were obtained from this case.

Unenhanced CT remains the most valued imaging modality in body packing, and is often used for confirmation when radiography or ultrasound is negative but a strong suspicion remains. Computed tomography allows not only confirmation, but also determines the size, number and localisation of the packages, in addition to any complications related to drug leaks (Figure 12a, b).^45^ The reported sensitivity and specificity of CT is between 96% – 100% and 94% – 100%, respectively, and the most widely accepted protocol is non-contrast CT without oral or rectal contrast, as these may hamper the intraluminal visualisation of the packets, which have similar density to contrast material. Computed tomography images should be assessed on both abdominal and lung windows, as trapped air in the packages is better seen with the lung window. On CT, packets containing solid drugs appear as multiple, oval-shaped, sharply margined structures of variable density in the bowel lumen with attenuation values ranging from -520 HU to 700 HU.^45^ Liquid narcotic containing packets tend to appear as homogeneously hyperdense structures with irregular shapes, taking the shape of the intestinal lumen, with attenuation values ranging from 155 HU to 310 HU. Low attenuating bands caused by air trapped between the package sheath and packages may be seen and are specific. In addition, a ‘jigsaw’ pattern has been reported resulting from packages interlocking with each other.

Although MRI may be used for evaluating suspected cases of body packing, its role is often regarded impractical because of cost, limited availability and longer examination time when compared with the other imaging modalities. Also, magnetic resonance (MR) artefacts secondary to motion can be intentionally introduced by a suspect, rendering the images suboptimal. Additionally, MRI can also be harmful to the body packer if the material used for packing the drugs contains ferromagnetic materials.^45^

Some of the fatal complications reported in the literature related to body packing include oesophageal, gastric or bowel obstruction or perforation, gastric ulceration and even respiratory arrest from aspiration of the package contents. The role of the radiologist does not stop with identifying and confirming the presence of concealed narcotics, but also extends to identifying the number, location of the packages and any signs of complications that can result from the rupture of these drug packets.^45^

### Foreign body aspiration

**FIGURE 13:**
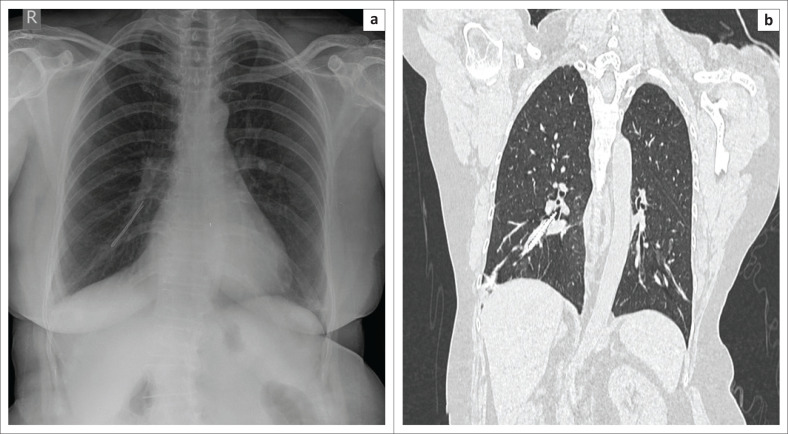
(a) Chest radiograph and (b) coronal reformatted chest computed tomography images of a 59-year-old lady who accidentally aspirated a hair pin (arrows) into the right lower lobe bronchus.

Foreign body aspiration is a rare entity in adults and is more commonly reported in children. Sehgal et al.^46^ conducted a systematic review of the literature on the subject of adult foreign body aspiration managed by flexible endoscopy. They reviewed the bronchoscopy database in PubMed for all studies in English from 1979 to 2014. A total of 25 998 flexible bronchoscopies were performed during this period, of which only 65 subjects (0.25%) had undergone bronchoscopy for foreign body aspiration. Imaging abnormalities were seen in 86.2% of the patients (*n* = 56) on chest radiography (CXR) or CT at the time of presentation and included non-resolving opacities, segmental atelectasis or lobar collapse, bronchiectasis or hyperinflation. The CXR was diagnostic in 16 patients (24.6%). Metallic foreign bodies (e.g. pins, whistles, etc.), followed by organic ones (e.g. betel nuts, peanuts, peas, rice, etc.), where the most common types of foreign bodies identified during bronchoscopy (Figure 13a, b). The right lower lobe bronchus (30.6%) was the usual site for foreign body aspiration. Unlike in children, who require rigid bronchoscopy for foreign body extraction, 90% of the aspirated foreign bodies in adults are managed by flexible bronchoscopy. Rigid bronchoscopy may be required in adults in certain circumstances such as failed flexible bronchoscopy attempt(s) to retrieve a foreign body, foreign bodies that are impacted in extensive granulation tissue or excessive tissue scarring, a large foreign body that cannot be gripped with flexible forceps, asphyxiating foreign bodies, foreign bodies with a smooth margin and sharp foreign bodies.^46^

## Conclusion

Imaging with plain radiography and MDCT, in particular, play a crucial role in the diagnosis and management of foreign bodies lodged in the aerodigestive tract. Familiarity with the common locations for impaction of these foreign bodies and their characteristic appearance on imaging combined with a cautious interpretational approach using multiplanar reformatted images and the knowledge to differentiate the common mimickers on imaging can aid in early and accurate diagnosis.
